# A pilot randomised controlled trial of negative pressure wound therapy to treat grade III/IV pressure ulcers [ISRCTN69032034]

**DOI:** 10.1186/1745-6215-13-119

**Published:** 2012-07-28

**Authors:** Rebecca L Ashby, Jo C Dumville, Marta O Soares, Elizabeth McGinnis, Nikki Stubbs, David J Torgerson, Nicky Cullum

**Affiliations:** 1 Department of Health Sciences, The University of York, York, UK; 2 Centre for Health Economics, The University of York, York, UK; 3 Leeds Teaching Hospitals NHS Trust, Leeds General Infirmary, Leeds, UK; 4 NHS Leeds Community Healthcare, St Mary's Hospital, Leeds, UK; 5 School of Nursing, Midwifery & Social Work, The University of Manchester, Manchester, UK

**Keywords:** Negative pressure wound therapy, Pressure ulcer, Pilot randomised controlled trial

## Abstract

**Background:**

Negative pressure wound therapy (NPWT) is widely promoted as a treatment for full thickness wounds; however, there is a lack of high-quality research evidence regarding its clinical and cost effectiveness. A trial of NPWT for the treatment of grade III/IV pressure ulcers would be worthwhile but premature without assessing whether such a trial is feasible. The aim of this pilot randomised controlled trial was to assess the feasibility of conducting a future full trial of NPWT for the treatment of grade III and IV pressure ulcers and to pilot all aspects of the trial.

**Methods:**

This was a two-centre (acute and community), pilot randomised controlled trial. Eligible participants were randomised to receive either NPWT or standard care (SC) (spun hydrocolloid, alginate or foam dressings). Outcome measures were time to healing of the reference pressure ulcer, recruitment rates, frequency of treatment visits, resources used and duration of follow-up.

**Results:**

Three hundred and twelve patients were screened for eligibility into this trial over a 12-month recruitment period and 12/312 participants (3.8%) were randomised: 6 to NPWT and 6 to SC. Only one reference pressure ulcer healed (NPWT group) during follow-up (time to healing 79 days). The mean number of treatment visits per week was 3.1 (NPWT) and 5.7 (SC); 6/6 NPWT and 1/6 SC participants withdrew from their allocated trial treatment. The mean duration of follow-up was 3.8 (NPWT) and 5.0 (SC) months.

**Conclusions:**

This pilot trial yielded vital information for the planning of a future full study including projected recruitment rate, required duration of follow-up and extent of research nurse support required. Data were also used to inform the cost-effectiveness and value of information analyses, which were conducted alongside the pilot trial.

**Trial registration:**

Current Controlled Trials ISRCTN69032034.

## Background

Negative pressure wound therapy (NPWT) was developed in the 1990s as a treatment for full thickness wounds such as severe pressure ulcers. NPWT involves the application of a suction force (negative pressure) across a wound surface via a dressing. It has been claimed that NPWT speeds wound closure, reduces infection rates and cuts labour costs [1-3]. Despite these claims, there is a growing recognition of a lack of high-quality research evidence to support the use of NPWT [4,5].

In their review, the United Kingdom (UK) National Health Service (NHS) Purchasing and Supply Agency was unable to draw conclusions about the relative effectiveness of NPWT for the treatment of any wound, and recommended that independent high-quality randomised controlled trials (RCTs) and economic evaluations be conducted [2]. There have been several other systematic reviews [6-8] and technology reports [9-12], all of which echo the need for research evidence regarding the clinical and cost-effectiveness of NPWT.

Whilst there is clinical uncertainty regarding the use of NPWT for most wound types, a key area of uncertainty is the clinical and cost effectiveness of NPWT in the treatment of severe pressure ulcers. Pressure ulcers (also called bed/pressure sores or decubitus ulcers) are a complication of acute illness and immobility, and affect between 4.7% and 32.1% of hospital patients at any point in time and between 4.6% and 20.7% of people in nursing homes [13]. Pressure ulcers range in severity from persistent hyperaemia of unbroken skin through to full thickness tissue necrosis down to bone, and at the time of this study, were graded from one to four (Table 1), with grades III and IV being severe. These wounds may take many months to heal, particularly where there is substantial loss of skin and muscle. Treatment strategies for these wounds include the use of pressure-relieving devices, wound dressings [14] and, more recently, NPWT. 

**Table 1:** **Pressure Ulcer Grading System (according to the European Pressure Ulcer Advisory Panel Grading System [**[15]**])**

Grade 1	Non-blanching erythema of intact skin. Discoloration of the skin, warmth, oedema, induration or hardness may also be used as indicators, particularly on individuals with darker skin
Grade 2	Partial thickness skin loss involving the epidermis, dermis or both. The ulcer is superficial and presents clinically as an abrasion or blister
Grade 3	Full thickness skin loss involving damage to or necrosis of subcutaneous tissue that may extend down to but not through the underlying fascia
Grade 4	Extensive destruction, tissue necrosis or damage to muscle, bone or supporting structures with or without full thickness skin loss

*At the time that this pilot trial was conducted, pressure ulcers were referred to as grades by the European Pressure Ulcer Advisory Panel. They are now classified as categories and stages, e.g. category/stage IV: full thickness tissue loss [16].

Pressure ulceration has been estimated to cost the UK between £1.4 and £2.1 billion per year, with hospital costs (including nursing care) being the key cost component [17]. One UK primary care Trust estimated that 33% of its NPWT-associated costs may be incurred for the treatment of severe pressure ulcers.

Whilst there is a need for further research evidence to reduce uncertainty regarding the value of NPWT in treating severe pressure ulcers, it would be premature to conduct a full trial, without assessing whether such a study is a worthwhile use of scarce research funding and feasible. Whether an RCT of NPWT for pressure ulcers would yield information of sufficient value to decision makers is the subject of a related paper [18]. This paper deals with the feasibility of such a trial. Large RCTs are challenging to design and expensive to implement. They may fail to reach their recruitment targets and may require funded extensions [19]. A multi-centre trial conducted in Canada comparing NPWT with standard care for the treatment of grade III/IV pressure ulcers was terminated prematurely because of poor recruitment [20]. Pilot trials are an important pre-requisite for identifying any potential problems that could impact upon a full trial [21,22] and are invaluable in informing study design, intervention, trial procedures, recruitment methods and resources, ensuring that these are feasible, acceptable and appropriate [22,23].

Our study had two aims. Firstly, to assess the feasibility of a full RCT of NPWT for severe pressure ulcers, we needed to pilot the design and methods. We were particularly keen to assess and/or test the following: rate of participant recruitment; data collection strategies; time to healing of grade III/IV pressure ulcers; spoken language of potential participants; rate of attrition (defined here as the loss of patients who did not formally exit the trial). We knew it was likely that participants would transfer across care boundaries between hospital and community settings; therefore the pilot would yield valuable information regarding likely rates of hospital admission and/or discharge and discharge destinations (as these have implications for the continuity of data collection).

Other methodological issues to be explored in the pilot included whether the point of healing could be collected with precision and whether it was feasible to blind the outcome assessor to treatment allocation. Finally, as nurses were responsible for completing trial documentation, we wanted to find out whether documentation used in the pilot was acceptable to nurses collecting the data as a task in addition to treating patients.

The second aim of this study was to collect additional trial data for use in a cost-effectiveness and value of information (VOI) analysis that was being conducted concurrently with the pilot trial. In order to inform these analyses, information regarding treatment use (frequency of dressing changes, length of treatment, discontinuation of treatment and reasons for treatment change), the occurrence and resolution of complications related to severe pressure ulceration (osteomyelitis and/or systemic infection), and the occurrence of pressure ulcer closure surgery.

The design of this pilot trial was informed by consultation with wound care nurses and wound care specialists. They agreed that spun hydrocolloid (hydrofibre), foam and alginate dressings were the relevant alternative treatments to NPWT in this patient population [24].

## Methods

### Research questions

The following research questions were formulated in order to assess the feasibility of conducting a larger trial:

a) Recruitment

· How many participants with grade III or IV pressure ulcers can be recruited to a trial of NPWT compared with standard care from acute and primary care in one city (population 715,000) over a 12-month recruitment period?

b) Data collection

· For how long should patients with grade III or IV pressure ulcers be followed up in order to capture healing? What are likely participant attrition rates and reasons for attrition?

· What are rates of hospital admission and discharge for trial participants, and what are their discharge destinations?

· How can healing data can be collected from trial participants and can healing be assessed by blinded assessors?

· How many potential participants will require translation of trial documentation?

· Can ulcer-related pain and health-related quality of life data be collected from trial participants?

· What are nurses’ views of the data collection documentation and burden?

In order to inform the second aim of the study (cost-effectiveness and VOI analysis), the trial was designed to also address the following research questions:

c) Treatment information

· How frequently are dressings changed for patients with grade III/IV pressure ulcers receiving NPWT and standard care?

· How long are patients with pressure ulcers typically treated with NPWT or standard care before they receive a change of treatment?

· What are the typical reasons for clinicians changing from NPWT or standard care to an alternative?

· What are the rates of typical adverse events in this population (specifically measuring any cases of osteomyelitis, systemic infection and ulcer closure surgery)?

### Study location, regulatory approval and patient consent

This was a multi-centre (acute and primary care providers within one city), randomised, controlled, fixed sample parallel group trial, using equal randomisation. Patients with pressure ulcers of grade III or IV who were receiving care within the boundaries of NHS Leeds (including those receiving community nursing care from Leeds Community Healthcare NHS Trust and hospital care from Leeds Teaching Hospitals NHS Trust) were screened against pre-trial inclusion and exclusion criteria for eligibility:

### Inclusion criteria

· Participants must have a pressure ulcer graded III or IV according to the European Pressure Ulcer Advisory Panel Grading System [15]

· Participants must receive primary care via Leeds Primary Care Trust (PCT)

· The pressure ulcer should contain at least 80% viable tissue or have a very thin layer of slough (nonviable tissue) requiring no further debridement prior to use of Negative Pressure Wound Therapy (NPWT) [25-28]

### Exclusion criteria

· Presence of unclear undermining in the pressure ulcer cavity, precluding the use of NPWT (i.e. the deepest point of ulcer cannot be measured)

· Pressure ulcer has necrotic tissue, eschar or necrotic bone present

· Patient has limited life expectancy, e.g. undergoing end-stage palliative care

· Pressure ulcer located where, in the opinion of the treating clinician, a vacuum seal cannot be obtained, e.g. the anus

· Pressure ulcer too close to exposed blood vessels and/or organs, anastomotic sites and/or nerves

· Patient is unable to give valid informed consent because of incapacity

· Patient is unable to consent as trial materials are not available in a suitable language*

· Patient does not wish to consent to participation within trial

· A clinical judgement has been made that the patient is not receiving adequate nutrition to allow treatment with NPWT

· Other reasons, in the clinical judgement of the treating clinician or nurse, which exclude the patient from the trial

*If selected, the location of the pressure ulcer, details of language requirements and/or other reason(s) for exclusion were recorded.

Written informed consent and verbal assent to participate within this study was obtained from each study participant. Ethical approval for this study was obtained from the Northern and Yorkshire Research Ethics Committee (reference no.: 08/H0903/22).

### Screening strategies

The research team developed multiple strategies to maximise the number of patients with pressure ulcers identified and screened:

a) A dedicated trial research nurse made enquiries to hospital ward staff to determine whether any patients had grade III/IV pressure ulcers.

b) A list of patients with pressure ulcers referred to the community Tissue Viability service (approximately 300) was made available to the research nurse.

c) The research nurse raised awareness about the study in the community setting. Tissue Viability nurses contacted the research nurse when they encountered patients with severe pressure ulcers.

The research nurse then screened potential participants from all these settings (community nursing services, hospital wards, nursing homes, residential homes) for their eligibility to participate in this trial.

### Baseline assessment

At recruitment, one pressure ulcer was deemed the reference ulcer that would be monitored for the duration of the trial. Where there was more than one eligible pressure ulcer, the deepest ulcer was defined as the reference ulcer. The research nurse completed a baseline assessment of each patient, recording demographic and clinical characteristics, details of which are shown in Table 2. Information regarding patient demographics, ulcer history and co-morbidities was obtained from patient notes by the research nurse. The grade and location of the reference ulcer was determined by visual inspection and patient notes. The baseline assessment also included measurement of the dimensions (length, width and depth) of the reference ulcer. Wound dimensions were measured to the nearest millimetre using a sterilised disposable flexible ruler. Length was defined as the length of the longest straight line and width as length of the longest straight line, perpendicular to the ulcer length line. Ulcer depth at the deepest point of the ulcer was measured using a sterilised disposable probe. Each ulcer was given an identification code (ID), and the shape and location of the ulcer drawn on a diagram of the body (front and back). At baseline, patients were also asked how intense the pain from their pressure ulcer(s) had been in the previous 24 h. This was plotted on a 10 cm visual analogue scale, which ranged from no pain to the worst pain imaginable and was measured to the nearest 0.5 mm using a standard 30-cm ruler by the research nurse. The patient’s treatment preference was recorded and participants were also asked to complete the EQ-5D health-related quality of life (HR-QoL) questionnaire.

**Table 2:** Patient data collected at baseline

**Patient demographics**	Date of birth, gender
	Current location of patient,
	Hospital speciality where patient was being treated (current inpatients only)
	Date of hospital admission (current inpatients only)
**Ulcer assessment and history**	**Ulcer assessment:**
	Grade of reference pressure ulcer
	Location of reference pressure ulcer
	Reference pressure ulcer dimensions (width, length and depth)
	**Ulcer history:**
	Duration of reference pressure ulcer
	Time since the development of first pressure ulcer
	Current number of grade I/II and III/IV pressure ulcers
**Infection**	Presence/absence of infection of the reference ulcer; if present, how infection was assessed
	Presence/absence of osteomyelitis
**Co-morbidities**	Presence of incontinence, cardiovascular disease, peripheral vascular disease, diabetes, arthritis, orthopaedic, airway, neurological stroke, cancer or other co-morbidities
**Treatment preference**	Indifferent to treatment allocation, prefer NPWT or prefer standard care
**Pain**	Pressure ulcer-related pain (24 hour recall) using a 10 cm visual analogue scale
**Quality of life**	EQ-5D questionnaire

### Randomisation

After completing the baseline assessment, the research nurse telephoned a secure and remote randomisation service, located at the York Trials Unit (University of York, UK). Randomisation was conducted using pre-generated random permuted blocks (block sizes of four and six). A data manager at the York Trials Unit, who was completely independent of the research team, created the randomisation programme, which was used to allocate participants to one of the two treatment arms: either NPWT or standard care (SC). Treatment was allocated on an individually named patient basis and participants commenced their allocated treatment immediately following randomisation. Participants were assigned an anonymised identification number, which was used to identify them throughout the trial.

Each participant’s General Practitioner was notified via letter of the patient’s involvement in this trial. A digital photograph was taken of the reference ulcer using a mobile camera phone (Sony Ericsson, C902 Cyber-Shot^TM^). The photograph included a depth probe in-situ and a card showing the patient ID number and the date the photograph was taken. The photograph was emailed directly from the camera phone to the York Trials Unit and stored on a secure server.

### Treatment details

a) NPWT

The NPWT devices used in this study were from the VAC Therapy Units and Systems range, manufactured by Kinetic Concepts Inc. (KCI) (San Antonio, TX, USA). Devices were used in accordance with the manufacturer’s guidance [25-27]. The duration of treatment was determined by the nurse treating the patient and also the patient, in accordance with current practice. The pressure ulcer being treated with VAC was filled with either VAC WhiteFoam® or GranuFoam™ dressings; other dressings or treatments/procedures were applied/performed as deemed necessary by the treating health professional. The type of VAC device, beginning and end date of NPWT treatment, any changes during the cycle, type of VAC foam, non-VAC dressings and any other treatment details or procedures were recorded at each treatment visit to the participant.

b) Standard care (SC)

Participants in the SC arm received one of the following, chosen by the treating nurse: a spun hydrocolloid (fibrous hydrocolloid) dressing, a foam dressing or an alginate dressing (all non-silver).

The frequency of dressing changes was determined by the nurse (standard practice). The type of trial dressing, reason for choice of dressing, type of secondary dressing applied (if applicable) and any other treatment details or procedures were also recorded at each treatment visit to the participant.

c) Non-trial treatment

In some cases, participants were no longer able to receive their allocated trial treatment and instead received a non-trial treatment (these participants remained in the trial). The reasons for treatment change and the type of treatment applied were recorded; the non-trial treatment applied was at the discretion of the treating clinician.

### Patient progress and data collection

The progress of participants through this trial and a summary of trial data collection processes are shown in Additional file 1 and Tables 3, 4 and 5. Table 6 shows the data collected during this pilot trial, and the feasibility aspect of a full trial and cost-effectiveness and VOI analyses, which these data informed. 

**Table 3:** Data collection process for recruitment data collected during the trial

**Data process**	**How and when collected**
Number of participants with grade III or IV pressure ulcers who can be recruited to a trial of NPWT compared with standard care from acute and primary care in one city (population 715,000) over a 12-month recruitment period	During pre-trial screening on pre-trial screening forms

**Table 4:** Data collection processes for outcome data collected during the trial

**Data process**	**How and when measured**
Length of follow-up for trial participants	The length of time each group participated in the trial was calculated as the difference between date of randomisation and date of trial exit
Frequency of attrition and reasons for attrition	Recorded if participant was lost to follow-up on the participant event form
Rates of hospital admission and discharge destinations	Date and details of inpatient hospital admission and discharge ecorded as they occurred and recorded on the participant event form
Collection of healing data from trial participants	The date the research nurse considered the reference ulcer to have healed was recorded on the participant event form. The time to healing was calculated as the difference between the date of randomisation and the date of healing. Once the reference pressure ulcer was considered to have healed, photographs were taken on the day of healing and then once per week for 3 weeks (so four healing photographs in total)
Blinded outcome assessment of wounds	At the end of the trial, a Tissue Viability Clinical expert (blinded to participant treatment allocation) visually inspected baseline, monthly and healing photographs of the reference pressure ulcer. For each series of photos, the assessor was required to state whether they considered the reference pressure ulcer to have healed, the treatment allocation and whether the quality of the photograph was adequate to make a decision about healing
Number of participants who would require translation of trial documentation	During pre-trial screening on pre-trial screening forms
Measurement of ulcer-related pain at dressing change and health-related quality of life data	Ulcer-related pain was measured on a 10-cm visual analogue scale that ranged from no pain to the worst pain imaginable. This was recorded on treatment monitoring forms pre- and post-application of dressing
	Participants were asked to complete the EQ-5D HR-QoL questionnaire at baseline and 2 weeks, 1, 3 and 6 months post-randomisation. Any difficulties participants had with completing these were noted by the research nurse
Nurses’ views data collection	Face-to-face and telephone meetings took place with the research nurse who would provide feedback from nurses on any problems they encountered with completing trial documentation

**Table 5:** Data collection processes for treatment data collected during the trial

**Data process**	**How and when measured**
Frequency of dressing changes in both trial arms	This was calculated from the number of treatment monitoring forms completed by the research nurses indicating a dressing change and included forms completed both prospectively and retrospectively
	Such data were collected retrospectively when a participant changed location or was self-caring. In such instances, the research nurse would consult patient notes or records kept by the patient to determine the number of dressing changes over a specified period of time
Duration of treatment with allocated trial treatment	Calculated as the difference between the date a patient receiving their allocated treatment to the date they stopped receiving this treatment. This information was available from treatment monitoring forms (as described above)
Reasons for ceasing allocated trial treatment	When, in the clinical judgement of the nurses, a patient could no longer receive their allocated trial treatment, the reason for treatment change was recorded on a treatment monitoring form
Define common adverse events in the study population	All serious and non-serious adverse events that occurred in participants were recorded on adverse events forms. The nature, description and relationship of the event to trial treatment were recorded. The research nurse indicated whether the event was considered to be related to the reference pressure ulcer

The data processes that were devised to answer the research questions formulated to meet the objectives of the study are shown, as are how and where these data were collected.

**Table 6:** Trial data and data collected

**Data collected in pilot trial**	**Aspect of full trial and/or cost effectiveness modelling and value of information analysis this informs**
Number of participants recruited over 12 months	Number of participating centres required
	Duration of recruitment period
	Eligibility criteria
Time to healing of participants’ pressure ulcers	Duration of follow-up required and sample size
Number of participants who withdraw from the study or die before study completion or who are lost to follow-up	Initial sample size
	Whether attrition is likely to threaten the validity of a future trial (due to attrition bias)
Reasons for withdrawal	Strategies to anticipate and minimise withdrawals
Rates of hospital admission, discharge, destinations	Which settings should be involved and how to ensure continuity of data collection
Photographs of pressure ulcers	Whether camera phones can be used to monitor healing in real time and whether photographs are adequate for blinded outcome assessment
Time of ulcer healing	Can assessment of healing be undertaken by blinded assessors from photographs?
Preferred language of (potential) participants	What proportion of future participants is likely to require translation of documentation into which languages?
Pain and health-related quality of life	Are the visual analogue scale (VAS) and EQ-5D questionnaire suitable in this population?
Nurses’ views of trial documentation	How can we optimise the data collection for a full trial to ensure completion by NHS staff?
Frequency of dressing changes	Resource use and structure of data collection tool
Time to change to treatment/nature of change to treatment and reasons	Resource use and structure of data collection tool
Adverse events (rates and nature)	Cost effectiveness and future data collection

Participants were followed up for a maximum of 6 months. Reasons for trial exit were: withdrawal of consent, loss to follow-up or death. Whilst the research nurse was primarily responsible for data collection, this responsibility was also delegated to nurses treating patients in acute and community settings.

### Clinical events

a) Healing

As the primary outcome measure of a full RCT is likely to be time to healing, data were collected on the date of complete healing (defined as epithelialisation and cessation of treatment to achieve healing) of the reference pressure ulcer. Since assessment of wound healing is somewhat subjective and staff and participants could not be blinded to treatment, it would be important to conduct blinded outcome assessment in a full trial. We piloted a blinded outcome assessment process using digital photographs of the wound taken using the mobile camera phone (Table 6). Photographs were taken every month by the research nurse and then weekly for 4 weeks once the ulcer had healed. Each photograph was emailed directly from the camera phone to the trials unit. A blinded outcome assessor (experienced Tissue Viability nurse not involved in the day-to-day conduct of the trial) then assessed photographs for (1) assessment of healing, (2) threats to blinding and (3) picture quality.

b) Adverse events

An adverse event is any untoward medical occurrence in a trial participant, regardless of whether it is regarded as having been caused by the trial treatment. As an integral part of monitoring the safety of participants throughout this trial, all serious and non-serious adverse events that occurred in participants during the trial were recorded. The following were classified as serious adverse events: death, any life-threatening risk, hospital admission/prolongation of hospitalisation, persistent or significant disability/incapacity and any other medically important condition [29]. We further defined the following important medical conditions as serious adverse events: inability of participant to provide on-going consent, systemic infection/sepsis and osteomyelitis. All other adverse events such as wound infection, skin damage, pain or formation of new pressure ulcers were regarded as non-serious adverse events. These data were used to define the common adverse events occurring in the study population and also provided information on the number of cases of osteomyelitis, systemic infection and closure surgery that occurred during the trial.

### Statistical methods

This pilot study was not designed to detect a treatment effect and therefore descriptive statistics were used to summarise data. Quantitative data are presented as whole numbers (*n*), proportions and/or percentages (%). Continuous data are presented as the mean ± standard deviation (SD) and/or the median and 25^th^ to 75^th^ centiles.

## Results

### Recruitment

Patients were screened for eligibility into this trial during the 12-month recruitment period (1 September 2008 until 31 August 2009); the trial ended on 31 October 2009. Whilst we originally planned a 6-month follow-up period, we extended the recruitment phase of the trial to maximise recruitment; therefore duration of follow-up varied between 2 months to a maximum of 6 months.

**Figure 1:**
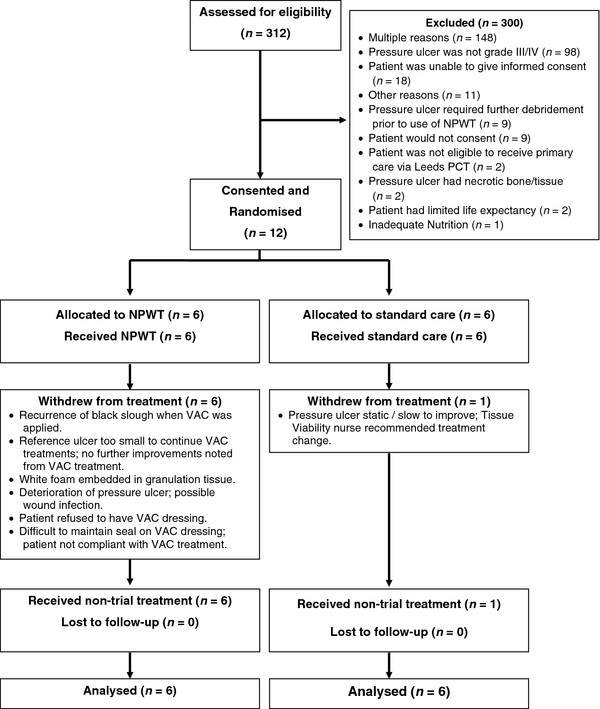
Flow of participants through the trial.

The flow of participants through this trial is shown in Figure 1. In total, 312 patients with pressure ulcers were screened for eligibility. Of these, 12 people were eligible for recruitment and all gave informed consent; 6 participants were randomised to NPWT and 6 to SC (conversion rate from screening to recruitment: 3.8%, 12/312). Of the 300 excluded patients, 152 (51%) had a single reason for exclusion (the remaining 49% had multiple reasons). The most common single reason for exclusion was a pressure ulcer less severe than grade III (98/152, 65%) followed by the patient being unable to give informed consent (18/152, 12%).

The baseline demographic and clinical characteristics for all trial participants are shown in Table 7. The most common location of reference pressure ulcers in the NPWT group was the sacrum, followed by the buttocks/gluteal region. No reference pressure ulcers were regarded as infected and a total of 39 co-morbidities were recorded at baseline. A greater number of co-morbidities occurred in the NPWT group; the most common co-morbidity in both groups was faecal incontinence (Table 8). 

**Table 7:** Baseline demographics and clinical characteristics of trial participants

**Characteristic**	**Result**
Gender (*n*)	5 male, 7 female
Age*	67.5 (54.5 to 82.0) years
Care setting (*n*) and patients location	3 acute: general ward (*n* = 3)
	9 community: patient’s home (*n* = 8), nursing home (*n* = 1).
Reference ulcer grade	Grade III (*n* = 7)
	Grade IV (*n* = 5)
Reference ulcer location:	
Heel (*n*)	1
Trochanter (*n*)	1
Sacrum (*n*)	5
Buttocks/gluteal (*n*)	3
Ischial (*n*)	2
Pressure ulcer dimensions:	
Width*	3.0 (2.0 to 3.8) cm
Depth*	4.0 (2.2 to 6.5) cm
Length*	5.0 (4.0 to 7.2) cm
Duration of reference ulcer*	4.0 (2.2 to 28.5) months
Time elapsed since development of first pressure ulcer*	24.0 (3.0 to 144.0) months
Number of patients with an infected reference ulcer (*n*)	0
Number of patients with osteomyelitis (*n*)	0
Number of patients who had a previous pressure ulcer at the same location as the current reference ulcer (*n*)	3
Number of co-morbidities (*n*)*	2.5 (2.0 to 5.0)
Number of patients with grade I/II pressure ulcer(s) in addition to the reference pressure ulcer (*n patients*)	0 grade I/II (*n* = 2 patients)
	1 grade I/II (*n* = 5 patients)
	3 grade I/II (*n* = 1 patient)
	Not recorded (*n* = 4 patients)
Number of grade III/IV pressure ulcer(s) in patients (*n patients*)	1 grade III/IV pressure ulcer (*n* = 8 patients)
	2 grade III/IV pressure ulcers (*n* = 2 patients)
	3 grade III/IV pressure ulcers (*n* = 1 patient)
	4 grade III/IV pressure ulcers (*n* = 1 patient)
Treatment preference	Prefer NPWT (*n* = 3)
	Prefer SC (*n* = 1)
	No preference (*n* = 8)
Number of patients able to complete the visual analogue pain scale (*n*)	9
Visual analogue pain scale score*	0.6 (0.2 to 3.9) cm

*Data are presented as median and 25^th^ to 75^th^ centiles.

**Table 8:** Baseline frequency of co-morbidities in trial participants

	**NPWT (*n*** = **6)**		**SC (*n*** = **6)**
**Co-morbidity**	**Frequency(*n*)**	**Co-morbidity**	**Frequency(*n*)**
Faecal incontinence	4	Faecal incontinence	3
Myocardial infarction	3	Multiple sclerosis	2
Non-insulin dependent diabetes mellitus	3	Spinal injury	2
Angina	2	Urinary incontinence	1
Heart failure	2	Other cardiovascular disease	1
Rheumatoid arthritis	2	Insulin-dependent diabetes mellitus	1
Chronic obstructive pulmonary airways/pulmonary disease	2	Femoral neck fracture	1
Stroke	2	Chronic obstructive pulmonary airways/pulmonary disease	1
Urinary incontinence	1	Other neurological	2
Ischaemia (leg/foot)	1	Myocardial infarction	0
Insulin-dependent diabetes mellitus	1	Angina	0
Multiple sclerosis	1	Heart failure	0
Other	1 (lymphoedema)	Ischaemia (leg/foot)	0
Other cardiovascular disease	0	Non-insulin-dependent diabetes mellitus	0
Femoral neck fracture	0	Rheumatoid arthritis	0
Spinal injury	0	Stroke	0
Other neurological	0	Other	0
**Total co-morbidities**	**25**	**Total co-morbidities**	**14**

Co-morbidities are ranked in order of descending frequency in each treatment group.

### Data collection

#### Duration of follow-up for trial participants in each group and frequency and reasons for attrition

The mean duration of follow-up in the NPWT group was 123 days [median = 125 (25th to 75th centiles = 68 to 185 days), minimum = 46 and maximum = 190 days], which was shorter than the mean for the SC group of 152 days [median = 169 (25th to 75th centiles = 130 to 188 days), minimum = 68 and maximum = 190 days]. One participant’s reference pressure ulcer healed during this trial; the time to healing was 79 days. There was no attrition recorded during this trial. From the 12 recruited participants, 5 (2 NPWT and 3 SC participants) exited the trial after completing the maximum 6-month follow-up. A further five participants (two NPWT and three SC) completed less than the 6-month follow-up as they were recruited later into the study. Two participants died during the trial; the official causes of their deaths were not recorded for the purposes of this study; however from the serious adverse event forms, the nurse reported that both participants had infections (chest infection and urinary tract infection) and their health was reported as generally deteriorating prior to death.

#### Rates of hospital admission and discharge and discharge destinations

Three community-based participants receiving SC were admitted to, and discharged from, hospital: a total of four admissions and six discharges were recorded. Destination after hospital discharge was recorded for one participant only. There were no hospital admissions or discharges recorded for the three community-based NPWT participants. No hospital discharges were recorded for NPWT patients who were hospital inpatients at baseline. Where participants moved between community and hospital care settings it proved impossible within the resources of the trial to continue the collection of contemporaneous data.

#### Collection of healing data from trial participants and blinded outcome assessment of reference pressure ulcer

All four scheduled healing photographs were taken of the one ulcer that healed during the pilot. After reviewing participants’ digital photographs, the blinded outcome assessor thought that 9/12 (75%) participants had not healed, was unsure whether 2/12 had healed and agreed with nurses that one participant had healed. In terms of the success of blinding, the assessor was unable to identify the treatment group in 10/12 (83%) of cases. In the remaining 2/12 cases the assessor rightly identified one participant as having been allocated to NPWT and wrongly identified another as having NPWT (as they believed foam marks were visible on the skin). In most cases (10/12, 83%) the assessor thought the picture quality was adequate to make an assessment of wound healing. In the remaining two cases the assessor noted that the wound was in the shadow of the buttock or deep in the natal cleft, so could not be fully certain that the wounds were unhealed.

#### Number of participants who would require translation of trial documentation

Pre-trial screening data showed 10/312 (3.2%) patients required trial documentation in a language other than English, these languages being Polish, Punjabi and Urdu.

#### Measurement of ulcer-related pain and collection of HR-QoL data from participants

The feasibility of collecting pre- and post-treatment pain data from participants was also assessed. Pain data could not be collected from three trial participants (3/12: 25%). These participants were unable to complete pain VAS scales because of impaired/lack of sensation below the waist caused by spinal injury or pathology. Nurses also reported that some patients had difficulty in understanding the concept of the visual analogue pain scale and suggested a different type of pain scale be used.

All EQ-5D HR-QoL questionnaires were completed, with the following two exceptions: one participant, who was confined to a wheelchair, modified question 1 (Mobility) of the EQ-5D questionnaire to read: “I am confined to bedwheelchair”. One participant was unresponsive following an epileptic seizure and the research nurse partially completed the EQ-5D questionnaire on behalf of the participant (collection of EQ-5D data by proxy has not been validated).

#### Nurses’ views of data collection documentation

A number of issues were identified by nurses with regards to the completion of trial documentation. Whilst a dedicated trial nurse was primarily responsible for data collection, in some instances, nurses were required to complete paperwork regarding dressing changes. This task was in addition to their usual duties and patient care. They reported that the large volumes of paper-based documentation to be completed at every patient visit was repetitious. In general, there was some informal feedback that some nurses were not clear as to why the research was being conducted; that the extra data collection was burdensome and that some professions felt their personal expertise was sufficient in determining treatment plans for participants.

Nurses also reported that treatment-monitoring forms were not designed to capture delays in participants receiving their allocated trial treatment or retrospective data. In some cases, a participant’s allocated treatment was not immediately available, so in the interim, participants were treated with whatever treatment was on-hand.

### Treatment information

#### Frequency of dressing changes, duration of treatment with allocated trial treatment and reasons for ceasing allocated trial treatment in both arms

There were a total of 1,236 treatment visits recorded during this trial. There were fewer trial treatment visits in the NPWT group [mean ± SD = 25 ± 23; median (25^th^ to 75^th^ centiles) = 14 (9 to 46)] than in the SC group [mean ± SD = 113 ± 56; median = 98 (64 to 173)]. The duration of trial treatment was also lower for NPWT (NPWT mean ± SD = 44 ± 45 days; median (25^th^ to 75^th^ centiles) = 22 (14 to 84) days vs. SC mean ± SD = 137 ± 59 days; median (25^th^ to 75^th^ centiles) = 156 (70 to 188)]. Overall, there were fewer treatment visits per week for patients receiving NPWT group compared with SC (NPWT = 3.1 vs. SC = 5.7).

Whilst all six NPWT participants moved to non-trial treatment, only one SC participant did so. The reasons for withdrawal from trial treatment are provided in Figure 1. Overall, the mean number of treatment visits whilst on non-trial treatment was similar in both groups (NPWT = 6.5 vs. 7.0 for SC).

#### Common adverse events in the study population

A total of 28 adverse events were reported in nine participants (five NPWT and four SC) (Table 9,10 and 11). Once case of osteomyelitis was reported and there were no cases of systemic infection or closure surgery during the trial. 

**Table 9:** Adverse events according to treatment group

**Type of adverse event**	**Treatment group**	
	**NPWT**	**SC**
**Serious:**	-	-
Death	2	-
Admission to hospital	1	3
Other medically important condition	-	1
Persistent/significant disability/incapacity	-	-
Life-threatening risk (immediate risk of death)	-	-
Participants cannot provide on-going consent	-	-
Systemic infection/sepsis	-	-
Osteomyelitis	1	-
**Non-serious adverse events:**	-	-
Wound infection	2	1
Skin damage	-	1
Wound bleeding	-	-
Breakdown of wound following closure surgery	-	-
Pain	1	-
Recurrence of pressure ulcer (grade II or above) at site of reference pressure ulcer	-	-
New pressure ulcer (grade II or above)	-	-
Other event*	9	6
**Total**	**16**	**12**

*Details of these are provided in Table 10.

**Table 10:** Details of non-serious adverse events classified as ‘other’ and research nurses’ classification as to whether the event was considered to be related to the reference pressure ulcer

**Events considered to be related to the reference pressure ulcer:**	**(*n*)**
Deterioration of reference pressure ulcer	**3**
Increase in size/depth of reference pressure ulcer	**2**
Unable to remove Versafoam from wound*	**1**
Review of adverse event: removal of Versafoam*	**1**
Patient fell over VAC tubing and was unable to get off floor*	**1**
Increase in bleeding, wound malodorous and macerated to wound border	**1**
**Events considered to be unrelated to the reference pressure ulcer:**	**(*n*)**
Chest infection	**2**
Urinary tract infection	**2**
Generally unwell	**2**
Review of adverse event (Versafoam in ulcer)*	**1**

*These non-serious adverse events were also considered to be definitely related to the trial treatment, as shown in Table 11.

**Table 11:** Adverse events and their relationship to trial treatment

**Serious adverse events:**						
	**Unrelated**	**Unlikely to be related**	**Possibly related**	**Probably related**	**Definitely related**	**Not able to assess if related**
Death	2	0	0	0	0	0
Admission to hospital	4	0	0	0	0	0
**Total**	**6**	**0**	**0**	**0**	**0**	**0**
**Non-serious adverse events:**						
Wound infection	0	1	2	0	0	0
Pain	1	0	0	0	0	0
Other event	5	5	1	0	3	0
**Total**	**6**	**6**	**3**	**0**	**3**	**0**

## Discussion

This pilot study yielded invaluable information regarding the feasibility of conducting a large-scale trial of NPWT for the treatment of severe pressure ulceration.

### Recruitment

Participant recruitment into this trial was difficult. Even though this study was conducted within the area served by one large Primary Care Trust (NHS Leeds is responsible for the healthcare of more than 715,402 residents [30]) and a number of strategies were devised to maximise the number of patients screened for eligibility into this study, the conversion rate from screening to recruitment was low compared to other wound care trials that we have conducted [31-33]. This is a particularly challenging population to recruit from, and a similar multi-centre trial conducted in Canada, comparing NPWT with standard care for the treatment of grade III/IV pressure ulcers, was unable to recruit their target sample size of 184 participants and was terminated prematurely [20].

If a full trial were to proceed in this patient group, it is clear from this pilot trial that reaching the required sample size would be problematic. Recruitment could be increased by modifying the inclusion criteria and/or increasing the number of trial sites.

The most frequent single reason for participant ineligibility within this trial was that pressure ulcers were of a lower severity than grade III. It is not appropriate to use NPWT as a treatment for pressure ulcers less severe than grade III [16,34]. We would suggest that any protocol for a full trial should include a re-screening strategy to re-assess patients with grade I/II pressure ulcers in case the severity has increased.

Furthermore, given the number of people who were ineligible for inclusion as they were unable to provide consent, proxy assent by a relative might be considered in a future trial [35]. However, a previous pressure ulcer trial found that relatives were less likely to give consent for trial participation than patients themselves [36].

A large number of trial sites would be required and/or a recruitment period longer than the 12 months piloted here to reach target recruitment in a full trial. Dependant on the number of potential centres within the UK, it is possible that an international multi-centre trial would be required [18]. However, such a trial would be costly to deliver.

### Data collection and trial design

The follow-up period of 6 months used in this pilot was insufficient as only one healing event was captured during this time. Since we embarked on this pilot study, data on wound-healing trajectories for grade III/IV pressure ulcers have been published based upon 211 participants from eight randomised controlled trials [37]. These data differ from ours and suggest patients with grade III/IV pressure ulcers entered into clinical trials would achieve 90% healing after 18 weeks; however it would take more than 2 years for all patients to heal completely. The cost effectiveness and VOI work conducted alongside this pilot, also using its data (described in detail elsewhere [18]), also suggest that a 2-year follow-up would give the best expected net benefit of sampling (that is, would prove the most value for money), though it may be difficult to complete follow-up for this length of time in patients with severe pressure ulcers, who are generally unwell and suffer from multiple co-morbidities.

A number of issues regarding data collection from participants and data collection tools and approaches used by nurses were brought to light during this study. Whilst the pilot was only able to include English readers/speakers, there were no participants excluded solely because trial documentation was not available in a suitable language. However, the use of document translation should be considered for each trial site, acknowledging any particular language issues known to local investigators.

The pilot provided useful information regarding the collection of ulcer pain data: nurses reported that they thought the VAS was not appropriate for participants in this study as they found it difficult to understand and could not complete it themselves. Patients with pressure ulcers are likely to be older and thus may have conceptual issues in using the VAS to rate pain and the VAS is associated with a high failure rate in older people [38-40]. The British Geriatrics Society and British Pain Society Guidelines recommend Verbal Rating or Verbal Descriptor and Numerical Rating scales are better tools for quantifying pain intensity in the older adult [38]. The inability to collect pain data from participants is likely to be an issue for a full trial, given that 25% of participants in this trial were unable to complete pain scales. Data collection tools would need to be designed in order to collect the reasons why pain data cannot be recorded.

This trial showed it was possible to use a camera phone for the collection of wound photographs, which were sent back to the York Trials Unit in real time, thus allowing overdue photographs to be chased up and facilitating data collection. Blinded outcome assessment was also shown to be feasible for assessing pressure ulcer healing. In this study, the blinded assessor’s assessment of wound healing and treatment allocation was accurate 75% and 83% of the time, though assessment of healing was hampered by the location of the wound in two instances. This problem could have been overcome by modifying photography protocols for wounds in specific areas of the body and/or the additional use of transparency tracings of the wound [41]. Due to the small number of participants within this study, it was not possible to carry out statistical analysis of the intra-observer agreement between blinded assessment of healing and treatment allocation.

The completeness of EQ-5D data suggests that it was acceptable for use in participants with pressure ulceration, thus confirming the findings of others [42]; however there may be some issues with the wording of the questionnaire when completed by participants who are wheelchair users. This has been found previously by Malley et al. [43] who also discussed changing the wording of the mobility section with the developers of the EQ-5D questionnaire (EuroQol group). However they report EuroQol would not allow any such changes to be made to the questionnaire.

With regards to the issues raised by nurses about data collection, the large number of dressing changes (and hence accompanying trial documentation required to be completed at each visit) could not have been envisaged at the start of the trial. In a full trial we would consider the use of web-based/electronic rather than paper-based data collection methods, which may increase the efficiency of data collection as well as provide ‘real-time’ data [44-46].

### Treatment information

This pilot, in a short space of time, successfully provided important participant data about the number of dressing changes and treatment visits; these data (along with data from literature reviews and expert opinion [24] regarding the use of treatments for severe pressure ulceration) were used to inform a cost-effectiveness and VOI analyses [18]. The cost-effectiveness results from this suggested whilst NPWT could cost less and be more effective in comparison to other treatments, the decision to use NPWT remains highly uncertain; thus this treatment may not, in fact, be cost-effective. It is also important to note that the VOI analysis suggested a three-arm trial comparing spun hydrocolloid dressing vs. NPWT vs. alginate dressing is the trial design that would provide the most value for money. This analysis also suggested that foam was unlikely to be a cost-effective treatment and thus should not be evaluated [18].

This trial also yielded information regarding duration of treatment with trial interventions. Notably all six participants allocated to receive NPWT moved to non-trial treatment during the course of the study. These participants also received their allocated trial treatment for less time compared to those in the SC group. Regular changes of treatments are common in wound care resulting from: response or non-response of the wound; patient views regarding the treatment; treatment cost and the plethora of different treatment options that are available to health professionals. Thus movement from trial to non-trial treatments is more common (and indeed expected) in wound care trials than in many other areas. For example, in the VenUS II trial approximately 40% of participants changed from trial to non-trial treatment [31].

In practice (and as emphasised in this pilot) NPWT is likely to be just one in a succession of treatments that people with a grade 3 or 4 pressure ulcer could receive. Thus if the results of trials in this area are to be meaningful to practice they will need to be pragmatic, acknowledging that it is likely to be the policy of having NPWT as an available treatment on a pathway of care that is being evaluated [47,48].

## Conclusions

The feasibility of a future RCT of NPWT for grade III/IV pressure ulcers depends in large part on the availability of sufficient numbers of eligible participants; such a trial would require many centres, possibly internationally.

The pilot study has been invaluable in enabling us to provide a number of judgements and recommendations with regards to improving recruitment and data collection, appropriate outcome measurement, trial design and safety reporting:

· Given the low rates of recruitment, a future study in this patient group will require a large number of centres.

· Patients with grade I/II ulcers should be re-screened at a later date as they may become eligible for inclusion.

· Proxy assent should be considered in order to recruit patients who lack the capacity to consent.

· Strategies for following the patient journey across boundaries of care should be developed (and resourced) to ensure continuation of data collection.

· Instruments such as the Verbal Descriptor and Numerical Rating scales should be used (rather than the VAS) for recording ulcer-related pain, and documentation should record instances where pain cannot be recorded in participants.

· Camera phones can be used for the collection of wound data, which can be sent back to the main research sites in real time to facilitate data retrieval.

· Blinded outcome assessment of digital photographs for the assessment of healing may be supplemented by the use of wound-tracing grids to ascertain wound healing.

· Web-based or electronic methods of data capture may increase data collection efficiency; ensure these methods of data capture are piloted by nurses responsible for data collection to inform feasibility and acceptance.

· Anticipate that some participants may be self-caring and an approach will need to be devised in order to collect treatment data from such participants. Consider the use of treatment ‘logs’, in which the patient records the treatments they apply. Ensure nurses responsible for data collection assess wound progress at pre-specified intervals as patients are unable to provide an informed clinical judgement of wound progress.

· A large volume of data will be generated if every dressing change is recorded. Consider recording instead when a treatment regimen changes and the reason for treatment change, rather than repetitious data collection of the same treatment for the same participant over a long period of time.

## Abbreviations

HR-QoL: Health-related quality of life; ID: Identification code; KC: Kinetic Concepts Inc; NHS: National Health Service; NPWT: Negative pressure wound therapy; RCT(s): Randomised controlled trial(s); SC: Standard care; SD: Standard deviation; UK: United Kingdom; VOI: Value of information.

## Competing interests

The authors declare that they have no competing interests.

## Authors’ contributions

RA coordinated the study, drafted and finalised the manuscript. JD designed the study and data collection tools and helped to draft the manuscript. MS participated in the design of the study and data collection tools and helped to draft the manuscript. EM, NS and DT participated in the design of the study and data collection tools. NC conceived of the study, was Chief Investigator and participated in the design of data collection tools and helped to draft the manuscript. All authors read and approved the final manuscript.

## Supplementary Material

Additional file 1 Participant progress and data collection tools used during the trial. Click here for file
