# Supplementary material for: A pilot randomised controlled trial of negative pressure wound therapy to treat grade III/IV pressure ulcers [ISRCTN69032034]
Source: Trials. 2012 Jul 28;13:119. doi: 10.1186/1745-6215-13-119 (PMC3533804; doi:10.1186/1745-6215-13-119)
Supplement: Additional file 1 — Participant progress and data collection tools used during the trial. [file 1745-6215-13-119-S1.doc]

**Appendix 1: Participant progress and data collection tools used during the trial.**

**Participant change of location** recorded in *Change of location Form*

**Digital photographs** of the reference ulcer at monthly intervals until healing or trial exit

**Participant questionnaires** (EQ-5D and ulcer depth)

*Participant questionnaire: 2 weeks*

*Participant questionnaire: 1 month*

*Participant questionnaire: 3 months*

*Participant questionnaire: 6 months*

**Pre-trial Screening**

*Pre-trial screening form*

**Eligible Patient**

*Patient Information Sheet*

**Consent and randomisation**

*Consent Form*

*Baseline Record Form*

Digital photograph of reference pressure ulcer

Participant’s GP informed of participant’s involvement in the study (*GP information Sheet*)

**Allocated to NPWT**

All Treatment visits recorded in *Phase 1: Treatment Monitoring Forms*.

**Allocated to Standard Care**

All Treatment visits recorded in *Phase 1: Treatment Monitoring Forms*.

**Participant no longer receives allocated treatment**

- Reason for treatment change recorded in *Phase 1: Treatment Monitoring Form*.

Participant receives non-trial treatments.

- All Treatment visits recorded in *Phase 2: Treatment Monitoring Form*.

**Trial exit**

The reason for trial exit was recorded in the Participant *Event Form*

**Participant events:** Ulcer healing, limb amputation, closure surgery, hospital discharge, change to data collection recorded in *Participant event Form*

**Adverse Events:** Serious and non-serious adverse events recorded and reviewed in *Adverse Event Form*

**Recorded throughout trial**

**Trial exit**
